# Characterization of Cucurbit Aphid-Borne Yellows Virus (CABYV) from Passion Fruit in Brazil: Evidence of a Complex of Species within CABYV Isolates

**DOI:** 10.3390/v15020410

**Published:** 2023-02-01

**Authors:** Andreza H. Vidal, Cristiano Lacorte, Marcio M. Sanches, Dione M. T. Alves-Freitas, Emanuel F. M. Abreu, Bruna Pinheiro-Lima, Raul C. Carriello Rosa, Onildo N. Jesus, Magnólia A. Campos, Gustavo P. Felix, Ana Clara R. Abreu, Yam S. Santos, Ana Luiza M. Lacerda, Arvind Varsani, Fernando L. Melo, Simone G. Ribeiro

**Affiliations:** 1 Embrapa Recursos Genéticos e Biotecnologia, Brasília 70770-917, DF, Brazil; 2 Instituto de Ciências Biológicas—IB, PPG BIOMOL, Universidade de Brasília, Brasília 70910-900, DF, Brazil; 3 Embrapa Gado de Corte, Campo Grande 79106-550, MS, Brazil; 4 Embrapa Agrobiologia, Seropédica 23891-000, RJ, Brazil; 5 Embrapa Mandioca e Fruticultura, Cruz das Almas 44380-000, BA, Brazil; 6 Centro de Educação e Saúde, Universidade Federal de Campina Grande, Cuité 58175-000, PB, Brazil; 7 Instituto de Ciências Biológicas—IB, Universidade de Brasília, Brasília 70910-900, DF, Brazil; 8 Departamento de Microbiologia, Instituto de Biotecnologia Aplicada à Agropecuária, Universidade Federal de Viçosa (UFV), Viçosa 36570-900, MG, Brazil; 9 The Biodesign Center for Fundamental and Applied Microbiomics, Center for Evolution and Medicine, School of Life Sciences, Arizona State University, Tempe, AZ 85287, USA

**Keywords:** polerovirus, CABYV, CABMV, mixed infection, *Passiflora*

## Abstract

High-throughput sequencing (HTS) has been an important tool for the discovery of plant viruses and their surveillance. In 2015, several virus-like symptoms were observed in passion fruit (PF) plants in Bahia state, Brazil. Using HTS technology, bioinformatics tools, RT-PCR, and Sanger sequencing, we identified the cucurbit aphid-borne yellows virus (CABYV, *Polerovirus*, *Solemoviridae*) in co-infection with cowpea aphid-borne mosaic virus (CABMV, *Potyvirus*, *Potyviridae*) in PF, in green manure, and spontaneous plants in several localities in Bahia. Complete genomes of CABYV-PF isolates were determined and analyzed with other CABYV isolates available in GenBank that have been identified in various countries. Phylogenetic analysis and pairwise identity comparison with CABYV isolates showed that CABYV-PFs are more closely related to French and Spanish isolates. Overall, analyses of all the CABYV genomes revealed that these could represent ten distinct species, and we thus proposed reclassifying these CABYV as isolates into ten species, tentatively named “*Polerovirus curcubitaeprimum”* to “*Polerovirus curcubitaenonum*”, and “*Polerovirus melo*”. CABYV-PF is a member of “*Polerovirus curcubitaeprimum*”.

## 1. Introduction

Passion fruit is a tropical plant (genus *Passiflora*, family Passifloraceae) cultivated in several regions of the world, Brazil being the largest producer of yellow passion fruit (*Passiflora edulis* Sims). Virus diseases are a common problem in passion fruit crops and are responsible for significant reductions in the yield and quality of the fruits.

Several viruses have been identified in passion fruit plants in Brazil. Outbreaks of various begomoviruses (genus *Begomovirus*, family *Geminiviridae*) have been reported in passion fruit fields in different regions of the country [1,2,3,4]. Other viruses, such as grapevine virus A (GVA, genus *Vitivirus*, family *Betaflexiviridae*) [5], passion fruit yellow mosaic virus (PFYMV, genus *Tymovirus*, family *Tymoviridae*) [6], cucumber mosaic virus (CMV, genus *Cucumovirus*, family *Bromoviridae*) [7], passion fruit green spot virus (PfGSV, genus *Cilevirus*, family *Kitaviridae*) [8,9], purple granadilla mosaic virus (PGrMV, unclassified) [10], passion fruit chlorotic mottle virus (PCMoV, genus *Citlodavirus*, family *Geminiviridae*) [11,12], and lettuce chlorosis virus (LCV, genus *Crinivirus*, family *Closteoviridae*) [13] have also been identified in this crop.

Despite the emergence of these viruses, the most serious outbreaks of virus diseases in passion fruit in the country have been associated with cowpea aphid-borne mosaic virus (CABMV, genus *Potyvirus*, family *Potyviridae*). CABMV is transmitted by diverse aphid species, including *Aphis gossypii* and *Myzus persicae*, and causes passion fruit woodiness disease (PWD) in Brazil [14,15]. Nonetheless, in 2015, a high incidence of severe virus-like symptoms (mosaic, yellowing, blisters, leaf, and fruit deformations) was observed in several passion fruit fields in Bahia, Northeast Brazil. Considering these symptoms, the possibility of infection by viruses other than CABMV was raised. Hence, we explored the viruses present in these plants by using high-throughput sequencing (HTS) technology as a detection method.

This analysis revealed the occurrence of cucurbit aphid-borne yellows virus (CABYV, genus *Polerovirus*, family *Solemoviridae*), hitherto not known to infect passion fruit, in mixed infection with CABMV in passion fruit plants collected in Lençóis and Jussiape, Bahia state [16]. CABYV was first identified and characterized infecting melon and cucumber in France [17]. Since then, it has been detected in diverse cucurbit crops, broad bean, chickpea, and passion fruit in many regions in Europe, Asia, Africa, South and North America, and Oceania [18,19,20,21,22,23,24,25,26,27]. CABYV is transmitted by aphids such as *A. gossypii* and *M. persicae*, but not mechanically [17]. Recently, a recombinant CABYV isolate was characterized in melon plants in Brazil, which was shown to be vectored by the whitefly *Bemisia tabaci* [28].

In this study, we determined the full genome sequences of CABYV isolates from passion fruit plants from different regions in the state of Bahia in Brazil. The molecular characterization of these CABYV isolates and comparison to other CABYV sequences available in GenBank unveiled a complex of different species within CABYV, leading to a proposed regrouping of CABYV sequences within the *Polerovirus* genus.

## 2. Materials and Methods

### 2.1. Plant Material

**Figure 1:**
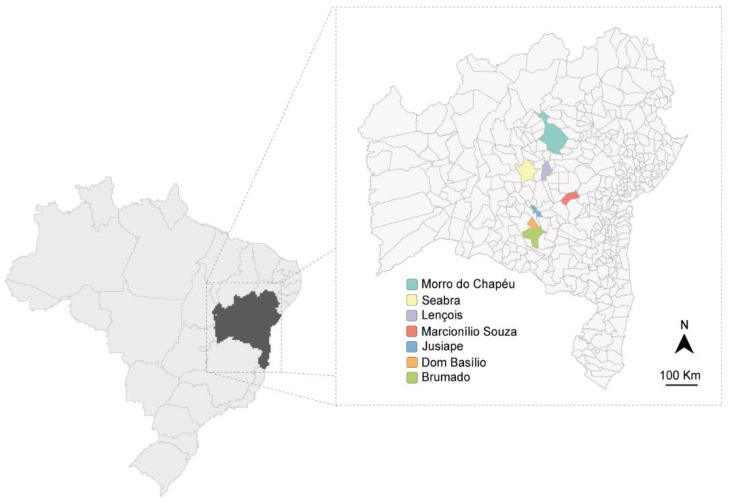
Map of Bahia state in Brazil showing the regions (in color) where plant material was collected.

Fifty-nine passion fruit samples (*Passiflora* spp.) were collected in June and July 2015 in different passion fruit fields in Bahia (Figure 1). The passion fruit plants exhibited severe virus-like symptoms (blisters, mosaic, yellowing, and leaf deformation) and were sampled in Marcionílio de Souza (*n =* 6 *P. edulis*), Seabra (*n =* 8 *P. edulis*), Morro do Chapéu (*n =* 2 *P. edulis*), Brumado (*n =* 2 *P. edulis*), Dom Basílio (*n =* 11 *P. edulis*), Lençóis (*n =* 21; *n =* 3 *P. cincinnata*, *n =* 3 *P. alata*, *n =* 15 *P. edulis*), and Jussiape (*n =* 9 *P. edulis*). In the same regions, several plants of different species used as green manure or spontaneous non-cultivated plants were also collected in Lençóis (*n =* 104), Dom Basílio (*n =* 25), Jussiape (*n =* 8), Seabra (*n =* 11), Marcionílio Souza (*n =* 12), Brumado (*n =* 38), and Morro do Chapéu (*n =* 5). All leaf samples were stored at −80 °C until analyzed. Preliminary results from the plants collected in Lençóis and Jussiape have been reported [16].

### 2.2. Nucleic Acid Extraction, High-Throughput Sequencing, and Data Analysis

Double-stranded RNA (dsRNA) and total RNA were extracted from the passion fruit plants (*n =* 59), according to Vidal et al. [13].

DsRNA aliquots (4 μL from each sample) were used to create two pools for the sequencing, according to Vidal et al. [13]. Pool PM1BA (*n =* 29) was composed of samples from Marcionílio de Souza, Seabra, Morro do Chapéu, Brumado, and Dom Basílio, while samples from Lençóis and Jussiape constituted pool PM2BA (*n =* 30). To achieve the necessary amount of RNA for sequencing (100 μg in total), 10 μL of total RNA of samples 502 and 594 were added in PM1BA, and 10 μL of total RNA of samples 724 and 581 in PM2Ba.

The libraries were prepared using Illumina TruSeq Stranded Total RNA and Ribo-Zero Plant kits and sequenced on an Illumina HiSeq 2500 platform (Macrogen Inc., Seoul, Republic of Korea). Paired-end reads (100 bp) generated in the Illumina HiSeq were checked for quality using FastQC [29]. The sequencing adapters were removed and the low-quality reads were checked using Trimmomatic [30]. The paired-end reads were then submitted to de novo assembly to obtain contigs using SPAdes assembler [31] with *k-mer =* 64. Contigs [>200 nucleotides] were compared against the NCBI virus database using BLAST search tool.

### 2.3. Virus Detection and Sanger Sequencing

The detection of CABYV and CABMV in the individual samples was conducted by RT-PCR assays using SuperScript™ III One-Step RT-PCR System with Platinum™ Taq DNA Polymerase kit (Invitrogen, Carlsbad, CA, USA), aliquots of total RNA, and virus-specific primers described in the literature. For CABYV detection, we used the set of primers Modified-CE-9F (a primer modified from CE-9F)/CE-10R [32] that amplify ~600 nt corresponding to the complete CP (coat protein) and partial MP (movement protein) genes. For CABMV, the primers CABMVLNJP2492F/CABMVLNJP3373R [13] that amplify ~900 nt corresponding to partial genes HC-Pro/p3 were used. The characteristics and sequence of primers used in this report are summarized in Table S1.

All amplicons were visualized by electrophoresis in agarose gel stained with ethidium bromide (Invitrogen, Carlsbad, CA, USA). Amplicons of selected samples with the expected size were excised, gel-purified, cloned in the pCR™2.1-TOPO™ vector following the manufacturer’s instructions (Invitrogen, Carlsbad, CA, USA), and Sanger sequenced (Macrogen, Seoul, Republic of Korea). All sequences obtained were analyzed and assembled in Geneious Prime^®^ 2022.1.1 software.

### 2.4. Southern Blot of CABYV RT-PCR

To confirm the identity of RT-PCR products, we used Southern blot hybridization with a CABYV-specific probe. After electrophoresis, the amplicons were transferred onto a nylon membrane Hybond™-XL (GE Healthcare, Pittsburgh, PA, USA) with denaturation buffer (0.5 N NaOH, 1.5 M NaCl) following the manufacturer’s protocol. Blots were UV-crosslinked using a UV Stratalinker 1800 (Stratagene, San Diego, CA, USA). The probe consisted of a CABYV- CP/MP-derived fragment labeled with radioactive [α^32^P] dCTP using an Amersham™ Rediprime™ II DNA Labeling System kit (GE Healthcare, Pittsburgh, PA, USA). Hybridizations were carried out overnight at 65 °C. Signals were detected by exposing the blots for 24 h in Fujifilm Imaging Plate BAS-IP MS, and the images were generated on a Fujifilm FLA-3000 Scanner.

### 2.5. Classification of Spontaneous Plants Positive to CABYV and CABMV

Classification of virus-positive spontaneous plants was based on plant morphology and DNA barcode rbcL and matK genes, according to Fazekas et al. [33]. First, DNA extraction was done using CTAB method [34]. Then, the rbcL and matK genes were amplified with Taq DNA Polymerase, recombinant (Invitrogen, Carlsbad, CA, USA) using the sets of primers SI_For/SI_Rev and KIM 3F/KIM 1R [35] (Table S1).

All amplicons were visualized by electrophoresis in agarose gel stained with ethidium bromide (Invitrogen, Carlsbad, CA, USA). PCR products were excised from the gel, purified, and Sanger sequenced at Macrogen Inc. (Seoul, Republic of Korea). Sequences were assembled in Geneious Prime^®^ 2022.1.1. and analyzed using the BOLD Identification System website [36].

### 2.6. 5′ and 3′ End Method for Rapid Amplification of cDNA Ends (RACE)

Based on the CABYV PF-M2BA (MH257573) sequence obtained from HTS data of pool PM2Ba [16], CABYV-specific primers (Table S1) were designed and used in the RACE method to determine the 5′ and 3′ ends of CABYV isolates from passion fruit. The RACE method was performed according to Alves-Freitas et al. [37], Schuster et al. [38], and Nicolini et al. [39].

For the 5′ RACE, cDNA was synthesized with SuperScript™ III Reverse Transcriptase (Thermo Fisher Scientific, Waltham, MA, USA) using 5 μg of RNA and primer CABYVRACE581R (10 mM). cDNA obtained was treated with RNase H (USB, USA) and RNase A (Thermo Fisher Scientific, Waltham, MA, USA), and purified with PureLink™ Quick Gel Extraction Kit (Thermo Fisher Scientific, Waltham, MA, USA). A homopolymeric tail of deoxycytidine (dCTP) was added to the cDNA 3′ end using Terminal Deoxynucleotidyl Transferase, Recombinant (Promega, Madison, WI, USA). The cDNA was dialyzed with an MF-Millipore™ Membrane Filter, 0.025 µm pore size (Merck, Rahway, NJ, USA). To obtain 5′ end fragments, two PCR assays were performed. The first PCR reaction was done using a cDNA prepared with gene-specific primer 1- GSP1 (CABYVRACE581R), and the forward anchor primer AAP (Table S1). For the second PCR, reactions were done using aliquots of the first PCR as a template and primers GSP2 (CABYVRACE581R) and anchor forward primer AUAP (Table S1).

For the 3′ RACE, first, a poly-A tail was added to the RNA (5 μg) using the *Escherichia coli* poly (A) polymerase (New England Biolabs, Ipswich, MA, USA). Then, the cDNA was synthesized with SuperScript™ IV Reverse Transcriptase (Invitrogen, Carlsbad, CA, USA) and anchored primer M10PacIT50VN (Table S1). Finally, the cDNA was treated with RNase H and RNase A and used in the PCR reactions. To obtain 3′ end fragments, two PCR assays were made. The first PCR was done using the cDNA and the primers GSP1 CABYVRACE5063F and M10 (Table S1). In the second PCR, aliquots of the first PCR were used as a template with primers GSP2 CABYVRACE5365F and M10 (Table S1).

All PCRs assays described above were performed with LongAmp^®^ Taq DNA Polymerase (New England Biolabs, Ipswich, MA, USA). The final PCR products of ~400 bp (5′ RACE) and ~350 bp (3′ RACE) were gel-purified, cloned into pCR™2.1-TOPO^®^ vector (Life Technologies, Carlsbad, CA, USA), and sequenced by Sanger method (Macrogen, Seoul, Republic of Korea). Sequences were analyzed in Geneious Prime^®^ 2022.1.1 and used to design primers required to amplify the complete CABYV sequences.

### 2.7. Complete Sequence of CABYV from Passion Fruit

**Figure 2:**
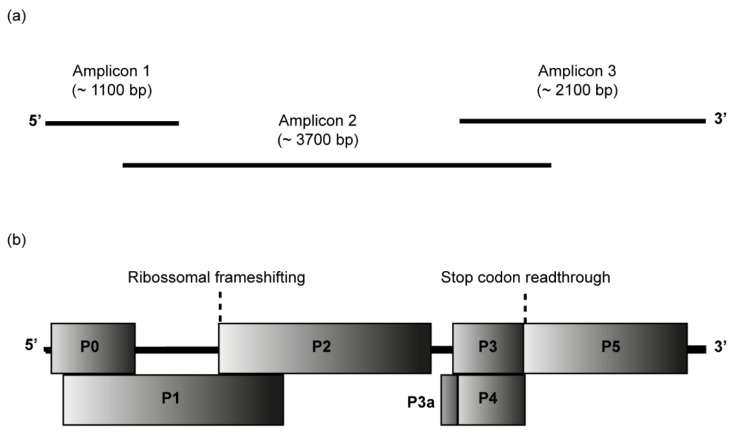
Passion fruit cucurbit aphid-borne yellows virus (CABYV) genome. (**a**) Amplicons 1 to 3 were cloned and sequenced to obtain the complete CABYV genome sequences from passion fruit. (**b**) Genome organization of the Brazilian CABYV from passion fruit showing the location of the predicted ORFs P0-P5 and P3a. ORF0 encodes the P0 protein, a putative RNA-silencing suppressor; ORF1 and ORF2 overlap and encode the P1-P2 protein which functions as RNA-dependent RNA polymerase; ORF3a encodes P3a protein, a systemic movement protein; ORF3 encodes P3 protein, the coat protein; ORF4 encodes the P4 protein, a long-distance movement protein; ORF5 is expressed by the suppression of the ORF3 stop codon to produce a CP-read-through domain (CP-RTD, P3-P5 protein), which is involved in the transmission by aphids.

The complete genome of CABYV isolates from passion fruit was determined by Sanger sequencing of overlapping RT-PCR products covering the entire genomes, as shown in Figure 2a. Passion fruit plants from Seabra (samples 558 and 564), Morro do Chapéu (sample 799), and Lençóis (samples 724, 726, and 729) were selected for full-length CABYV genome sequencing.

cDNA was synthesized with SuperScript™ III Reverse Transcriptase (Thermo Fisher Scientific, Waltham, MA, USA), total RNA, and reverse primer CABYV3R (Table S1). Three sets of primers (for amplicons 1, 2, and 3) were used to recover the full-length genome of CABYV in PCRs assays performed with KAPA HiFi Hotstart DNA polymerase (Roche Molecular Systems, Pleasanton, CA USA). All sets of primers used to amplify the amplicons 1, 2, and 3 are summarized in Table S1. CABYV5F and CABYV3R primers were designed based on sequences obtained by the RACE recovery of 5′ and 3′ ends. CABYV377F and CABYV1186R were primers derived from the MH257573 sequence [16]. Additional specific primers were from a previous report [32]. Amplicon 1 of ~1100 bp, amplicon 2 of ~3700 bp, and amplicon 3 of ~2100 bp were purified, cloned into the pJET 1.2/blunt vector (ThermoFisher Scientific, Waltham, MA, USA), and Sanger sequenced by primer walking (Macrogen, Seoul, Republic of Korea). Sequences were assembled in Geneious Prime^®^ 2022.1.1. BLASTn search was used to check the identities among sequences obtained in this research and other CABYV sequences in GenBank. Open reading frames (ORFs) were annotated using ORF Finder (accessible at https://www.ncbi.nlm.nih.gov/orffinder/, accessed on 1 August 2022).

### 2.8. Phylogenetic Analysis

A search in the GenBank database of the National Center for Biotechnology Information (NCBI) (accessible at https://www.ncbi.nlm.nih.gov/, accessed on 15 November 2022) for complete CABYV isolate sequences and viruses belonging to the family *Solemoviridae* was performed, and the sequences retrieved. Pairwise nucleotide and amino acid identity scores were calculated with Sequence Demarcation Tool (SDT) v1.2 [40]. MUSCLE [41] alignments generated in Geneious Prime^®^ 2022.1.1. were used to infer the maximum-likelihood (ML) phylogenetic trees using RAxML-NG v. 1.0.3 software [42]. The best-fit model TIM2+I+G4 was found for the ML phylogenetic tree using ModelTest-NG v0.1.7 [43]. The ML trees were calculated with 1000 bootstrap replicates and final trees were edited and visualized using FigTree v1.4.4 (accessible at http://tree.bio.ed.ac.uk/software/figtree/, accessed on 25 November 2022).

### 2.9. Recombination Analysis

Recombination analyses were performed with RDP4 v.4.100 software [44] with default settings, and a Bonferroni corrected p-value cut-off of 0.01. Analyses were conducted using RDP, GENECONV, BootScan, MaxChi, Chimaera, SiScan, and 3Seq methods, and only recombination events supported by at least three methods were considered.

## 3. Results and Discussion

### 3.1. HTS Data and Identification of CABYV and CABMV

High-throughput sequencing technology (HTS) has been an important tool for exploring the diversity of viruses in plants. In this research, an HTS approach allowed the identification of the RNA viruses CABYV (genus *Polerovirus*, family *Solemoviridae*) and CABMV (genus *Potyvirus*, family *Potyviridae*) in passion fruit library PM1Ba and confirmed the results for PM2Ba [16].

The two dsRNA passion fruit libraries sequenced by Illumina Hiseq 2500 resulted in 12,273,995 (PM1Ba) and 11,755,714 (PM2Ba) raw paired-end 100-bp reads. After trimming and processing, the reads were assembled into 104,931 contigs (72 to 12,062 nt in size) for PM1BA and 87,231 contigs (87 to 15,812 nt of length) for PM2BA.

All contigs (>200 nt) were analyzed by BLASTn against the viral NCBI database. Thirteen contigs of 203 to 485 nt and one of 5663 nt sharing >90% nt identities with CABYV were identified in the PM1BA and PM2BA libraries, respectively. Another fifteen contigs in PM1BA and thirty-nine in PM2BA were also identified, showing similarity with a potyvirus. These contigs ranged from 203 nt to 8458 nt in size, with 87% to 93% nt identity with CABMV.

Mapping all raw paired-end reads was performed on NCBI RefSeq genomes of CABYV (NC_003688) and CABMV (NC_004013). A total of 396 reads in PM1BA and 33,291 reads in PM2BA with >91% identity with the CABYV reference sequence were identified in the mapping. For CABMV, we identified 722,828 reads in PM1BA and 26,412,942 reads in PM2BA, with an identity of >97% regarding the reference genome.

### 3.2. Detection of CABYV and CABMV in Passion Fruit Plants

In this study, we identified a high incidence of CABYV and CABMV in the passion fruit samples. The presence of CABYV in passion fruit samples was evaluated by RT-PCR, followed by Southern hybridization with a CABYV-derived probe. Amplicons (~600 nt) of the expected size were visualized in the RT-PCR gel electrophoresis for several passion fruit samples. The amplicon identity was confirmed by positive hybridization with a CABYV-specific probe in the Southern blot (Figure S1).

In total, 21 (~36%) of the 59 tested passion fruit samples were identified as being infected with CABYV. CABYV was identified in five regions in Bahia (Figure S1; Tables S2 and S3). A high incidence of CABYV in the tested samples was recorded with 62.5% (5/8) of the plants in Seabra, 50% (1/2) in Morro do Chapéu, 27.2% (3/11) in Dom Basílio, and 11.1% (1/9) in Jussiape. In Lençóis, 52.3% (11/21) of the passion fruit plants were positive for CABYV, which was identified in at least one sample of three *Passiflora* species sampled in this region (01/03 of *P. cincinnata*; 02/03 of *P. alata*; 8/15 of *P. edulis*) [16]. On the other hand, plants from Marcionílio de Souza and Brumado were negative for CABYV.

Since CABMV is the most common virus infecting passion fruit, we tested the plants for its presence by RT-PCR (Figure S2). Initially, CABMV detection with CABMVM1MX_3726F/CABMVM1MX_5039R primers [11] was negative for some plants from Lençóis and Jussiape [16], despite these plants having exhibited typical symptoms induced by this potyvirus. This pair of primers are based on sequences of CABMV from Mato Grosso do Sul [11] and apparently were not suitable to screen for CABMV from Bahia (Northeastern Brazil). Indeed, RT-PCR using another set of primers, CABMVLNJP2492F/CABMVLNJP3373R, as described by Vidal et al. [13], confirmed the CABMV infection in the majority of the plants previously identified as negative when tested by Vidal et al. [16] with CABMVM1MX_3726F/CABMVM1MX_5039R primers.

As a result of this new round of analysis, CABMV was identified in 83.3% (5/6) of plants from Marcionílio de Souza, in 87.5% (7/8) from Seabra, 50% (1/2) from Brumado, in 72.7% (8/11) of the samples from Dom Basílio, in 71.4% (15/21) from Lençóis, and in all plants sampled in Morro do Chapéu (2/2) and Jussiape (9/9) (Figure S2a; Tables S2 and S3).

Mixed infections of CABYV and CABMV were also often identified in the passion fruit plants surveyed (Table S2). Both viruses were found in co-infection in 62.5% (5/8) of the samples from Seabra, in 38% (8/21) from Lençóis, 50% (1/2) from Morro do Chapéu, in 22% (3/11) from Dom Basílio, and in 11.1% (1/9) from Jussiape.

CABYV and CABMV cause several types of symptoms in their hosts. For example, infection by CABYV in cucurbits induces yellowing, thickening of older leaves, and a decrease in the number of fruits per plant, and the intensity of the symptoms can vary depending upon cultivar and other biotic and abiotic factors [17]. Infection by CABMV in passion fruit induces different levels of wrinkling, blisters, mosaic, deformation, and anatomical changes in the leaf, while the fruits can display woodiness and deformation] [45,46].

In this study, we were unable to associate CABYV with a particular symptom type. The CABYV-positive passion fruit plants exhibited similar symptoms, such as crinkling, mosaic, leaf and fruit deformation, blistering, yellow spot, chlorosis, yellowing, vein banding, green spot, vein whitening, and purplish leaf (Table S3). Most of these symptoms resemble those induced by CABMV. The major symptom usually associated with CABYV is leaf yellowing. Three *P. edulis* plants (samples 739, 732, and 716) from Lençóis were detected with CABYV in single infection, and showed symptoms of blistering, leaf deformation, mosaic, and vein banding (Table S3). In addition to the mixed infection of CABYV and CABMV in these plants, we have recently reported in sample 603 (*P. edulis*, Table S3) collected in Dom Basílio, a mixed infection of the crinivirus lettuce chlorosis virus and CABMV [13]. It is worth mentioning that in addition to RNA viruses, it is possible that these plants are also infected with DNA viruses.

CABYV seems to be disseminated in passion fruit producing areas in Bahia, Brazil. New screenings should be conducted in other producing areas to verify the dispersal of CABYV in Brazil. So far, preliminary results have indicated the occurrence of mixed infection of CABYV and CABMV in passion fruit plants from experimental fields in Rio de Janeiro, in the southeast of the country [47].

### 3.3. Detection of CABYV and CABMV in Spontaneous and Green Manure Plants and Classification of Positive Plants

Of all the spontaneous and green manure plants evaluated in this study, CABYV was detected only in plants from Lençóis. Of 104 spontaneous and green manure plants tested from Lençóis, seven were positive for CABYV infection (Figure S3). CABMV was evaluated only in the CABYV-positive plants (Figure S2b). The potyvirus was detected in three of the seven plants infected with CABYV.

These virus-positive plants were classified at the genus level. DNA barcoding with matK and rbcL genes was adopted to aid in classifying these plants (Table S4). Three PCR products of rbcL (544 nt) and matK (821 nt to 823 nt) sequenced from samples 540, 543, and 717 showed >99% similarity with *Macroptilium* spp. (genus *Macroptilium*, family Fabaceae). From sample 757, only the rbcL gene (544 nt) was sequenced and it was found to be 99.63% similar to the rbcL gene from *Stylosanthes* spp. (genus *Stylosanthes*, family Fabaceae). For samples 541, 760, and 761, the PCR products sequenced for rbcL (543 to 544 nt) and matK (811 to 826 nt) had 98.05% to 100% identity with *Cucumis* spp. (genus *Cucumis*, family Cucurbitaceae), *Sida* spp. (genus *Sida*, family Malvaceae), and *Bignonia* spp. (genus *Bignonia*, family Bignoniaceae).

It is known that viruses can spill over into cultivated plants from spontaneous plants, or from cultivated plants to spontaneous plant populations, which can lead to the emergence of new viruses or increase the host range of a virus [48,49]. We identified green manure and spontaneous plants as new hosts for CABYV and CABMV. Both viruses have a wide range of host plants and can infect cultivated and non-cultivated plants of diverse plant families. CABMV can infect plants of the families Passifloraceae, Fabaceae, Cucurbitaceae, Solanaceae, Chenopodiaceae, Amaranthaceae, and Poaceae [50,51,52,53]. CABYV has a wide range of Cucurbitacea plants as hosts and can infect several other plant families, such as Brassicaceae, Asteraceae, Malvaceae, Fabaceae, Amaranthaceae, Chenopodiaceae, Papaveraceae, Lamiaceae, Portulacaceae, Solanaceae, and Passifloraceae [16,17,20,27,32,54,55,56,57,58]. However, CABYV infection in *Macroptilium* spp., *Stylosanthes* spp., *Sida* spp., and *Bignonia* spp., as well as CABMV infection in *Sida* spp. and *Bignonia* spp., have not yet been reported. It is possible that these alternative host plants are natural reservoirs of CABYV and CABMV in Brazil.

### 3.4. Sanger Sequencing and Confirmation of CABYV and CABMV

Sanger sequencing of selected RT-PCR amplicons confirmed the identity of CABYV and CABMV in passion fruit, green manure, and spontaneous plants. CABYV-derived CP/MP amplicon sequences (600 nt) obtained from passion fruit plants from Seabra (sample 564; OP909788), Morro do Chapéu (sample 799; OP909789), Dom Basílio (sample 611; OP909791), Lençóis (samples 724, 726, 729, 738, 739; OP909790, and OP909792 to OP909795), Jussiape (sample 580; OP909796), as well as isolates from spontaneous and green manure plants (samples 540, 541, 543, 717, 757, 761, 760; OP909797 to OP909803) from Lençóis, had an nt identity of >99% amongst them. BLASTn search also revealed that these CABYV isolates shared the highest nt identity of 98% to 100% with sequence CABYV-PF-M2Ba (MH257573) [16]. In addition, CABYV isolates showed >94% nt identity with diverse sequences of CABYV available in GenBank.

CABMV HC-Pro/p3 fragments (904 nt) obtained from passion fruit from Lençóis (samples 524, 724; OP909781 and OP909783), Marcionilio de Souza (sample 500; OP909780), Brumado (sample 629; OP909782), and Morro do Chapéu (sample 799; OP909784), and of spontaneous plants (samples 717, 760, 761; OP909785 to OP909787), showed an nt sequence identity >93% among them. These isolates also shared 88% to 92.24% nt identity with the Brazilian CABMV MN124782 isolate from *Passiflora* spp. from Brazil.

### 3.5. Genome Characterization of CABYV from Passion Fruit

CABYV isolates from six passion fruit plants (Seabra: samples 558 and 564; Morro do Chapéu: sample 799; and Lençóis: samples 724, 726, and 729) were selected for further molecular characterization. The complete genome of the isolates, herein referred to as CABYV-PF, was determined by Sanger sequencing of three RT-PCR amplicons (amplicons 1 to 3), as schematized in Figure 2a. At least two clones of each amplicon were sequenced. In the complete genome assembly, the consensus sequence extracted from at least two clones of each amplicon was used. In the end, consensus sequences of amplicons 1 to 3 were considered for the final assembly.

In the sequence analysis of clones from samples 724 and 726, we observed that amplicons 1 and 2 (corresponding regions of ORF0 to ORF1) had several polymorphisms in the nucleotide sequence. These fragments with polymorphisms likely represent variants of CABYV in the same plant and did not interfere with P0 and P1 ORF prediction. For amplicon 3, few polymorphisms were observed for samples 724 and 726, and the sequences obtained from the other samples. Thus, based on the polymorphic sequences, we considered three sequences from sample 724 and two from sample 726 as variants of CABYV in these samples.

Nine full sequences of CABYV-PF isolates were obtained and characterized. The sequences from plants collected in Seabra (CABYV-PF-558 and CABYV-PF-564; OP909804 and OP909805), Morro do Chapeu (CABYV-PF-799; OP909806), and Lençóis (CABYV-PF-724-1, CABYV-PF-724-2, CABYV-PF-724-1, CABYV-PF-726-1, CABYV-PF-726-2, and CABYV-PF-729; OP909807 to OP909812) were deposited in the GenBank.

These isolates’ sequences ranged from 5672 nt to 5677 nt and showed a typical polerovirus genome organization (Figure 2b). The genomes have seven open reading frames (ORFs), a 5′ untranslated region (UTR) of 20 nt, a 3′ UTR that ranges from 161 to 163 nt, and between the ORF2 and ORF3a there is a non-coding internal region (IR) of 81 nt. All CABYV-PF isolates showed the same genomic organization, with minor differences only in the P3-P5 ORFs, which encode P3-P5 gene/fusion protein CP-RTD (coat protein-read-through).

ORFs 0, 1, and 2 are situated closer to the 5′ UTR. The ORF0 (P0 protein) overlaps the ORF1 (P1 protein) in 596 nts, and ORF1 overlaps the ORF2 in 547 nts. The ORF0 is 717 nt-long and encodes the P0 protein of 239 aa containing the conserved domains of the Luteo_P0 super-family and an F-box [59,60,61,62]. The ORF1 has 1893 nt and encodes the P1 protein of 631 aa. The P1 protein presents an aa sequence similar to the serine protease domain of the Peptidase_S39 family and virus protein genome-linked (VPg) typical of poleroviruses [63]. The ORF1 (1893 nt) and ORF2 (1275 nt) encode the P1-P2 protein (RNA-directed RNA polymerase—RdRp) of 1056 aa through -1 ribosomal frameshift mechanism due to a slippery sequence 5′-GGGAAAC-3′/5′-UUUCCC-3′ [63,64]. The P1-P2 protein displays the motifs typical of RdRps [60].

Closer to the 3′ UTR are localized the ORFs 3a, 3, 4, and 5. Located upstream of ORF3 (P3 protein), the ORF3a (P3a protein) has the translation initiated by an ATA codon driven by a Met-tRNA, resulting in an N-terminal methionine instead of an isoleucine [65,66]. The ORF3a has 138 nt and codes for a putative P3a protein of 46 aa. The ORF3 (P3 protein) codes for the coat protein (CP) that overlaps almost entirely with the movement protein (ORF4/P4 protein). The ORF3 of 600 nt encodes the P3 protein of 200 aa that presents the aa sequence of Luteo-coat super-family [67]. P3 protein (CP) of all CABYV-PF isolates have the aa sequence GILKAYHE typical of poleroviruses, which share the 5′-G[I/M]LK[A/S]YHE-3′ motif sequence [60]. The ORF4/P4 protein has 576 nt and encodes a putative movement protein of 192 aa. Located downstream of ORF3, the ORF5 is a translational in-frame read-through of the ORF3 stop codon to produce the P3-P5 protein, also known as fusion protein CP-RTD [60,61,62,63]. The P3-P5 was the sole protein that showed a difference in size among the CABYV-PF isolates, ranging in size from 2004 nt to 2013 nt, coding for a protein of 668 aa to 671 aa. The conserved proline-rich sequence 5′-PPPPGPSPT[P/-]P[P/S]PPPP-3′ typical of CABYV and other poleroviruses [60] was identified in P5 and was located just downstream of the CP stop codon. Two minor changes were observed in the proline-rich sequence in CABYV-PF799 (5′-PPPPGPSPT[-]PPPPPP-3′) and CABYV-PF729 (5′-PPPPGPSPTPP[S]PPPP-3′) isolates.

### 3.6. Phylogenetic Relation of the CABYV-PF Isolates

CABYV-PF isolates showed high similarities with CABYV isolates available in GenBank. BLASTn search showed that CABYV-PF (OP909804 to OP909812), as expected, shared the highest nucleotide identities of 94.39% to 99.50% with CABYV PF-M2BA (MH257573), a partial genome sequence determined from passion fruit and reported by our group [16]. High nt identities of 90.4% to 94.26% were also observed with CABYV sequences from France (MT027103, X76931, MZ202344) and Spain (JF939812-14, MW051362, MW051363).

As of November 2022, 56 complete CABYV sequences were available in GenBank. These sequences were determined from Cucurbitaceae and Solanaceae infected plants in localities of Republic of Korea (*n =* 31), Spain (*n =* 5), China (*n =* 6), France (*n =* 3), Brazil (*n =* 3), Taiwan (*n =* 2), India (*n =* 2), Papua New Guinea (*n =* 1), Japan (*n =* 1), the United States (*n =* 1), Indonesia (*n =* 1), and Timor-Leste (*n =* 1). CABYV complete sequences were retrieved from GenBank and compared with CABYV-PF isolates.

CABYV sequences retrieved from GenBank have been characterized as belonging to the genus *Polerovirus* into the family *Luteoviridae*. Recently, a new taxonomy was proposed, including *Polerovirus*, *Enamovirus*, *Polemovirus*, and *Sobemovirus* as genera belonging to the family *Solemoviridae* [63]. To better understand the relationship of the CABYV isolates to other solemoviruses, a phylogenetic tree was inferred based on the full-length sequences of CABYV isolates deposited in GenBank, CABYV-PF isolates from this study, and members of the *Solemoviridae*. In the phylogenetic tree, all CABYV isolates were grouped with members of the genus *Polerovirus*.

**Figure 3:**
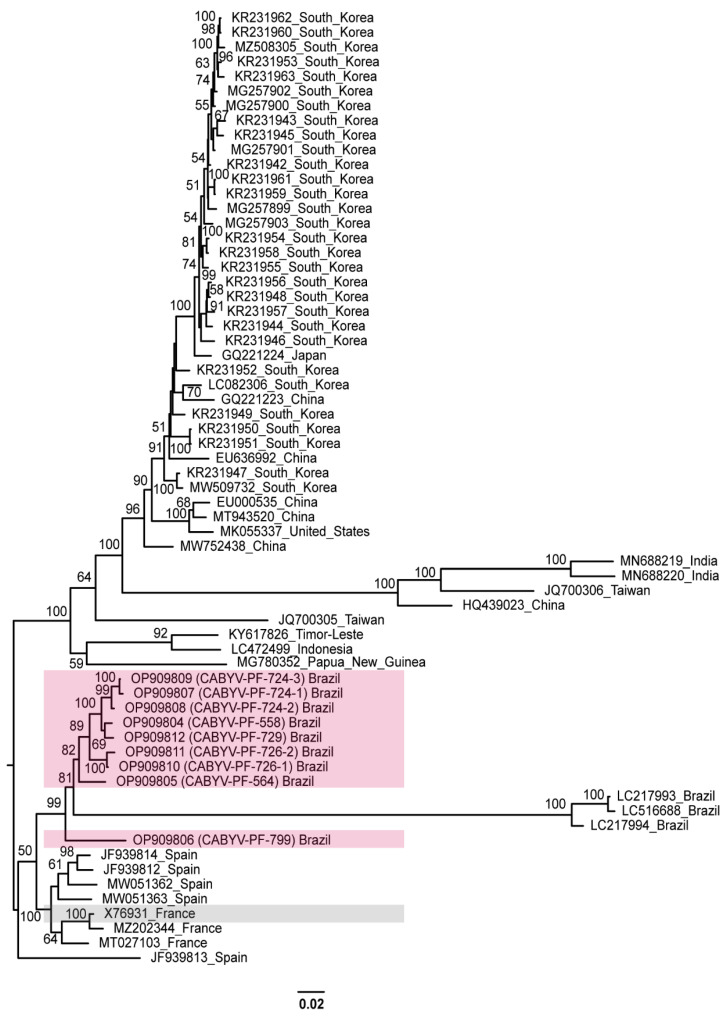
Midpoint-rooted phylogenetic tree of the complete nucleotide sequences of the CABYV sequences obtained in this study and of other isolates available in GenBank. Pink and gray boxes highlight CABYV-PF isolates and CABYV reference sequence, respectively. Phylogenetic trees were reconstructed using maximum likelihood in RAxML-NG v. 1.0.3 software using a MUSCLE alignment generated in the Geneious Prime^®^ 2022.1.1. Bootstrap support values >50% for 1000 replicates are displayed on or near the branches. The bar indicates 0.02 nucleotide substitutions per site.

A phylogenetic tree comprising CABYV-PF (OP909804 to OP909812) and all other CABYV sequences showed that the passion fruit isolates were most closely related to the Brazilian, French, and Spanish isolates (Figure 3).

Based on the evolutionary distance observed in this tree, there is a diversification for the other CABYV strains. Different clades were formed by sequences from Republic of Korea, China, Japan, the United States, India, Taiwan, Indonesia, and Timor-Leste. These groups were similar to the phylogroups described by Khanal et al. [18]. The distances were more evident for Spanish (JF939813) [32], Indian (MN688219 MN688220) [68], Chinese (HQ439023) [69], and Taiwanese (JQ700306) [70] isolates that were characterized as recombinants. The Brazilian isolates from melon (LC217993, LC217994, and LC516688), which are more related to CABYV-PF, were also identified as recombinants [22,28].

**Figure 4:**
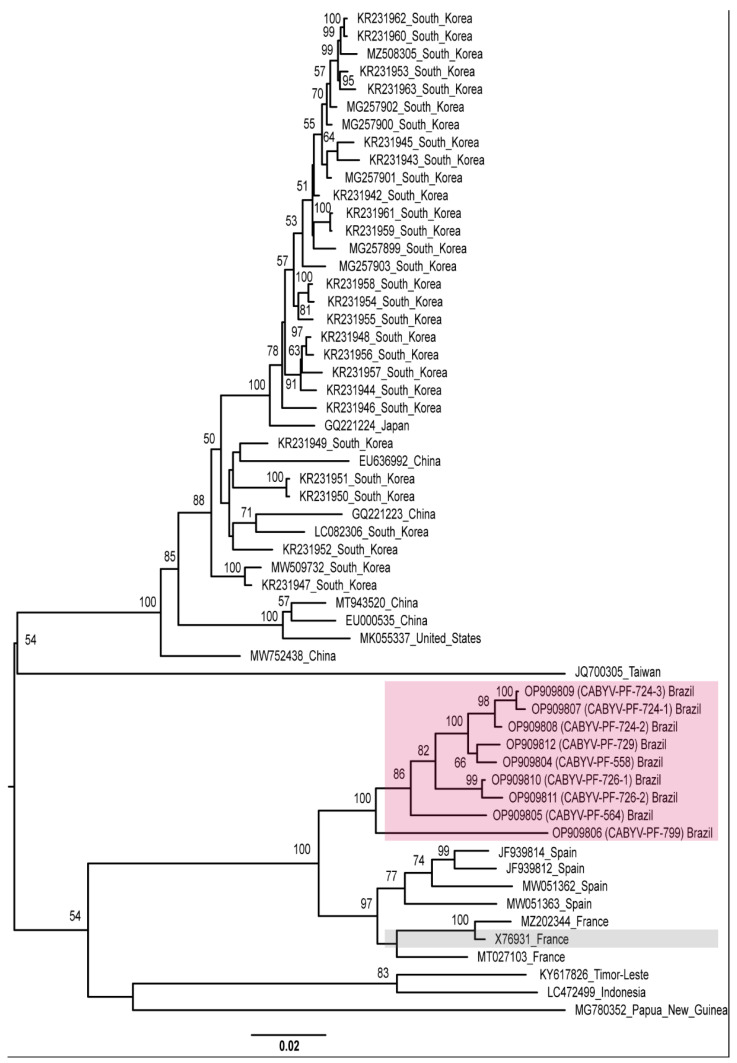
Midpoint-rooted phylogenetic tree of the complete genome sequences of CABYV sequences excluding CABYV recombinant sequences. Pink and gray boxes highlight CABYV-PF isolates and CABYV reference sequence, respectively. Phylogenetic trees were reconstructed using maximum likelihood in RAxML-NG v. 1.0.3 software using the MUSCLE alignment generated in Geneious Prime^®^ 2022.1.1. Bootstrap support values >50% for 1000 replicates are displayed on or near the branches. The bar indicates 0.02 nucleotide substitutions per site.

Recombination increases the genetic diversity of viruses [71]. Therefore, as an alternative view of the phylogenetic relationships of the CABYV isolates, we repeated the phylogenetic analyses excluding the recombinant sequences. Different groups were observed with isolates from different geographical regions, showing high genetic distances. CABYV-PF isolates still grouped with the French and Spanish isolates (Figure 4).

### 3.7. Relation between CABYV-PF Isolates and Brazilian Recombinant Isolates

Interestingly, CABYV isolates from melon from Brazil were more related to the passion fruit isolates. These isolates were characterized as recombinants between CABYV-N from France (X76931) and an unknown virus [22,28].

Analysis of the full-genome sequences revealed that CABYV-PF isolates were highly similar (94.3% to 96.7% nt identity) to the Brazilian melon isolates in the region that corresponds to 5′ UTR, P0, P1, P1-P2 (positions 1 to 3361 nt), up to the intergenic region, while the remainder of their genomes differed substantially.

Brazilian CABYV melon isolates were previously characterized as recombinants of CABYV-N from France as the major parent and an unknown minor parent [22]. Since we sequenced seven new genomes from Brazilian CABYV passion fruit isolates, we reassessed the recombination analysis, revealing that the CABYV melon isolates (LC217993, LC217994, LC516688) originated from a recombination event involving CABYV-PF-726-2, as the putative major parent, and a minor unknown parent (Figure S4). Our results support that the recombination event probably occurred in Brazil between CABYV common-type isolates, as those from passion fruit, and another unknown polerovirus, as hypothesized by Costa et al. [28].

We have investigated the possible presence of the CABYV recombinant type in the passion fruit plants evaluated in this study. The HTS data were reanalyzed, searching for the recombinant type, but no evidence was found. Mapping of all the reads using the Brazilian sequences from melon as references covered only ~60% of the genome (the non-recombinant portion). On the other hand, no read matched the recombinant region, represented by ~40% of the genome. Thus, we discarded the possibility of the recombinant type infecting these passion fruit plants. Likewise, Costa et al. [28] evaluated the occurrence of CABYV common-type in melon plants infected with the recombinant CABYV by RT-PCR with specific primers based on the CABYV-N (X76931) and CABYV-PF (MH257573) sequences with negative results.

We also ruled out that the CABYV isolates detected in green manure and spontaneous plants evaluated in this study are the Brazilian recombinant type. The portion of the CABYV genome (complete P3 [CP] and partial P4 [MP] genes) sequenced share an nt identity of about 64% with the recombinant type and >99% with the passion fruit isolates, i.e., common-type, and related to X76931. One of the alternative hosts we identified for the CABYV-common type was *Cucumis* spp. (Table S4). Since the Brazilian recombinant type also infects different *Cucumis* species [28], the recombination event that originated the Brazilian recombinant type likely occurred in a cucurbit plant, possibly a *Cucumis* species. Despite the negative results reported by Costa et al. [28], it is possible that CABYV-common type is infecting *Cucumis* or other cucurbits in producing fields, alone or in mixed infections with the recombinant type. Future surveys in cucurbits should address this issue.

Recombination analysis also revealed events of intraspecific recombination in CABYV-PF (Figure S4). However, as pointed out by Kassem et al. [32], these results should be taken with caution, considering that, apparently, this plant has a mixed infection with CABYV variants.

### 3.8. Amino Acid Pairwise Identity and Classification of CABYV Isolates

Phylogenetic relation (Figure 3 and Figure 4) based on the complete genome sequence of CABYV isolates from this study and those reported in previous studies revealed a diversification of CABYV. Currently, one of the species demarcation criteria for the genus *Polerovirus* is based on differences in amino acid sequence identity of any gene product of greater than 10% [55]. This criterion has been used to propose novel member species in the genus *Polerovirus* [72,73,74,75,76,77,78,79]. Accordingly, CABYV isolates that present an amino acid identity <90% in at least one protein regarding CABYV-N (RefSeq: NC_003688, GenBank: X76931), the first isolate identified in melon plants in France [17], would be considered as members of a different virus species. Therefore, based on the phylogenetic studies and the species demarcation criterion for the genus *Polerovirus*, we raised the question: Do the CABYV viruses reported in the literature fit this criterion?

Initially, we considered the non-recombinant sequences in the comparisons with CABYV-N from France (X76931). Pairwise identity analysis of the complete nucleotide sequence by Sequence Demarcation Tool (SDT) showed that CABYV-PF isolates share 77% to 94% identity with CABYV isolates previously reported. Analysis of individual deduced protein sequences (P0 to P5, and P3a) revealed that CABYV-PF isolates share an aa identity of 88.7% to 91.2% in P0; of 90% to 92.7% in P1; of 95.4% to 97% in P2; of 95.6% to 100% in P3a; of 97% to 99% in P3; of 95.3% to 96.9% in P4, and 93.6% to 95.1% in P5, compared to CABYV-N from France (X76931). Similar percentages between Spanish (JF939812, JF939814, MW051363, and MW051362) and French (MT027103 and MZ202344) isolates were also observed for all genes. All pairwise identities can be accessed in the supplementary file Table S5.

Among all CABYV isolates analyzed, P0 was the most divergent protein (Table S5), in agreement with LaTourrette et al. [80], which identified this genome region as hypervariable with high nucleotide diversity. P0 of CABYV-PF-799 (89.5%), CABYV-PF-724-1 (88.7%), and CABYV-PF-723-3 (89.1%) showed amino acid identity <90% regarding the French CABYV-N isolate (sequence X76931). However, these isolates share >90% amino acid identity with other CABYV-PF isolates (OP909804 to OP909812), which share >90% aa identity with the French isolate (sequence X76931). Thus, all CABYV-PF isolates should be considered strains of the same virus species.

Except for the Spanish (JF939814, JF939812, MW051363, MW051362) and French isolates’ sequences (MT027103, MZ202344), in the other CABYV isolates’ sequences, in the pairwise identity a difference >10% amino acid identity was observed in at least two proteins with respect to the French CABYV-N isolate (X76931) (Table S5). Overall, this difference could be perceived by taking into account P0 and P1. However, besides P0 and P1, other proteins also showed an identity difference of >10% in the aa sequence for some isolates. Considering the sequence divergence among all CABYV isolates (Table S5), and according to the mentioned species demarcation criterion for the genus *Polerovirus*, the CABYV isolates were grouped into different species. Isolates CABYV-PF, Spanish (JF939814, JF939812, MW051363, MW051362), and French (MT027103, MZ202344, and X76931) would be classified as members of the same species since they share aa identities >90% for all proteins. Other non-recombinant CABYV isolates would be grouped into at least four other species. The suggested classification for the cucurbit aphid-borne yellows virus can be found in Table 1.

The distinct species were renamed according to the new binomial nomenclature for virus species [85]. The species were tentatively named “*Polerovirus curcubitaeprimum*”, “*Polerovirus curcubitaesecundum*”, “*Polerovirus curcubitaetertium*”, “*Polerovirus curcubitaequartum*”, and “*Polerovirus curcubitaequintum*”, and the correspondent virus member names would be cucurbit aphid-borne yellows virus 1 to cucurbit aphid-borne yellows virus 5 (CABYV1 to CABYV5) (Table 1).

Based on the amino acid identity, all CABYV-PF from Brazil and the isolates from France and Spain were included in the proposed “*Polerovirus curcubitaeprimum*” species. Thirty-eight isolates from Republic of Korea, China, Japan, and the United States were included in the proposed “*Polerovirus curcubitaesecundum*” species. Isolates from Papua New Guinea (MG780352) and Taiwan (JQ700305) would be included as members of the species “*Polerovirus curcubitaequartum*” and “*Polerovirus curcubitaequintum*”, respectively. The isolates from Indonesia and Timor-Leste were included as members of the “*Polerovirus curcubitaetertium*” species, with the addendum that these two isolates differ only in the P0 (89.5% aa identity), while for the other proteins, these isolates share >90% amino acid identity, as seen in Table S5.

The recombinant isolates also were included in analyses, and thus five additional species were considered. Hence, the isolates from Taiwan (JQ700306), from India (MN688219 and MN688220), and from China (HQ439023) would be separated according to differences in P0, P1, and P2, while the isolate from Spain (JF939813) diverges in the P0 and P1 (Table S5). Indian isolates (MN688219 and MN688220) were designated as strains of the same species but diverged only in P0, sharing 89.2% amino acid identity, similar to the case of the Indonesian and Timorese isolates. The Brazilian recombinants (LC217994, LC217993, and LC516688) could be joined to compose a species according to differences seen in the P3a, P3, P4, and P5.

The Spanish, Taiwanese, Indian, Chinese, and Brazilian recombinant isolates were placed in the species named “*Polerovirus curcubitaesextum*”*,* “*Polerovirus curcubitaeseptimum*”, “*Polerovirus curcubitaeoctavum*”, “*Polerovirus curcubitaenonum*”, and “*Polerovirus melo*” (Table 1). The virus names proposed for the virus members of these species are cucurbit aphid-borne yellows virus 6 to cucurbit aphid-borne yellows virus 9 (CABYV6 to CABYV9). For the Brazilian isolates from melon, the name cucurbit whitefly-borne yellows virus (CWBYV) was suggested by Costa et al. [28].

According to our findings, the 56 complete sequences described as CABYV strains belong to a complex of polerovirus species and, along with the CABYV-PF identified in this study, would be classified in at least ten distinct species that infect mainly Cucurbitaceae, Solanaceae, and Passifloraceae plants (Table 1).

## 4. Conclusions

In this study, we obtained the complete genome of CABYV isolates from passion fruit and simultaneously categorized several strains previously identified as CABYV around the world as belonging to a complex of 10 different species in the genus *Polerovirus*. Members of these species infect mainly hosts in the families Cucurbitaceae, Solanaceae, and Passifloraceae. The passion fruit isolates were classified as members of the “*Polerovirus curcubitaeprimum*” species. We detected a high incidence of CABYV1 in mixed infection with CABMV in passionfruit plants, which can be an emergent problem in this fruit crop in Brazil. Further studies are needed to evaluate the epidemiology of CABYV1, the impacts of the interaction of mixed CABYV1/CABMV on crop productivity, and the identification of the insect vectors responsible for the transmission/establishment of these viruses. This information can provide a better understanding of the biology of CABYV and the measures needed to control the spread of CABYV in passion fruit crops in the country.

## Figures and Tables

**Table 1:** Suggested classification for the cucurbit aphid-borne yellows virus.

Species Name; Virus Name—Acronym	Country (State; City)	Host	Reference
GenBank Accession Number			
*Polerovirus curcubitaeprimum* Cucurbit aphid-borne yellows virus 1—CABYV1			
X76931	France	*Cucumis melo*	[17]
MT027103	France	*Cucumis melo*	Unpublished
MZ202344	France	*Physalis floridana*	Unpublished
JF939812	Spain	*Cucurbita pepo*	[32]
JF939814	Spain	*Cucurbita pepo*	
MW051363	Spain	*Citrullus lanatus*	[81]
MW051362	Spain	*Cucumis melo*	
OP909804 (CABYV-PF-558)	Brazil (Bahia; Seabra)	*Passiflora edulis*	This study
OP909805 (CABYV-PF-564)	Brazil (Bahia; Seabra)	*Passiflora edulis*	
OP909806 (CABYV-PF-799)	Brazil (Bahia; Morro do Chapéu)	*Passiflora edulis*	
OP909807 (CABYV-PF-724-1)	Brazil (Bahia; Lençóis)	*Passiflora edulis*	
OP909808 (CABYV-PF-724-2)	Brazil (Bahia; Lençóis)	*Passiflora edulis*	
OP909809 (CABYV-PF-724-3)	Brazil (Bahia; Lençóis)	*Passiflora edulis*	
OP909810 (CABYV-PF-726-1)	Brazil (Bahia; Lençóis)	*Passiflora edulis*	
OP909811 (CABYV-PF-726-2)	Brazil (Bahia; Lençóis)	*Passiflora edulis*	
OP909812 (CABYV-PF-729)	Brazil (Bahia; Lençóis)	*Passiflora edulis*	
*Polerovirus curcubitaesecundum* Cucurbit aphid-borne yellows virus 2—CABYV2			
MG257899	Republic of Korea	*Cucumis sativus*	Unpublished
KR231957	Republic of Korea	*Cucumis melo*	[61]
KR231942	Republic of Korea	*Cucumis melo*	
KR231943	Republic of Korea	*Cucumis melo*	
KR231944	Republic of Korea	*Cucumis melo*	
KR231945	Republic of Korea	*Cucumis melo*	
KR231946	Republic of Korea	*Cucumis melo*	
KR231947	Republic of Korea	*Cucumis melo*	
KR231948	Republic of Korea	*Cucumis melo*	
KR231949	Republic of Korea	*Cucumis melo*	
KR231950	Republic of Korea	*Cucumis melo*	
KR231951	Republic of Korea	*Cucumis melo*	
KR231952	Republic of Korea	*Cucumis melo*	
KR231953	Republic of Korea	*Cucumis melo*	
KR231954	Republic of Korea	*Cucumis melo*	
KR231955	Republic of Korea	*Cucumis melo*	
KR231956	Republic of Korea	*Cucumis melo*	
KR231958	Republic of Korea	*Cucumis melo*	
KR231959	Republic of Korea	*Cucumis melo*	
KR231960	Republic of Korea	*Cucumis melo*	
KR231961	Republic of Korea	*Cucumis melo*	
KR231962	Republic of Korea	*Cucumis melo*	
KR231963	Republic of Korea	*Cucumis melo*	
LC082306	Republic of Korea	*Cucumis melo*	Unpublished
MG257900	Republic of Korea	*Cucumis sativus*	
MG257901	Republic of Korea	*Cucumis melo*	
MG257902	Republic of Korea	*Cucumis melo*	
MG257903	Republic of Korea	*Citrullus lanatus*	
MW509732	Republic of Korea	*Cucumis melo*	
MZ508305	Republic of Korea	*Cucumis sativus*	
EU636992	China	*Cucumis melo*	
GQ221223	China	*Cucurbita pepo*	
MT943520	China	*Luffa cylindrica*	
MW752438	China	*Cucumis melo*	
GQ221224	Japan	*Cucumis sativus*	
EU000535	China	*Cucurbita moschata*	[82]
MK055337	United States	*Cucurbita maxima*	[83]
*Polerovirus curcubitaetertium* Cucurbit aphid-borne yellows virus 3—CABYV3			
LC472499	Indonesia	*Cucumis sativus*	Unpublished
KY617826	Timor-Leste	*Cucumis sativus*	[84]
*Polerovirus curcubitaequartum* Cucurbit aphid-borne yellows virus 4—CABYV4			
MG780352	Papua New Guinea	*Cucumis sativus*	[24]
*Polerovirus curcubitaequintum* Cucurbit aphid-borne yellows virus 5—CABYV5			
JQ700305	Taiwan	*Momordica charantia*	[70]
*Polerovirus curcubitaesextum* Cucurbit aphid-borne yellows virus 6—CABYV6			
JF939813	Spain	*Cucumis melo*	[32]
*Polerovirus curcubitaeseptimum* Cucurbit aphid-borne yellows virus 7—CABYV7			
JQ700306	Taiwan	*Luffa aegyptiaca*	[70]
*Polerovirus curcubitaeoctavum* Cucurbit aphid-borne yellows virus 8—CABYV8			
MN688219	India	*Cucurbita pepo*	[68]
MN688220	India	*Citrullus lanatus*	
*Polerovirus curcubitaenonum* Cucurbit aphid-borne yellows virus 9—CABYV9			
HQ439023	China	*Cucurbita pepo*	Unpublished
*Polerovirus melo* cucurbit whitefly-borne yellows virus—CWBYV			
LC217993	Brazil (Rio Grande do Norte; Mossoro)	*Cucumis melo*	[22,28]
LC217994	Brazil (Bahia; Juazeiro)	*Cucumis melo*	
LC516688	Brazil (Rio Grande do Norte; Mossoro)	*Cucumis melo*	

## Data Availability

Virus clones are available upon request. The sequences generated in this study are available at NCBI.

## Supplementary Materials

The following supplementary supporting information can be downloaded at: https://www.mdpi.com/article/10.3390/v15020410/s1, Table S1. Primers used in this study; Table S2. Number of passion fruit plants positive for CABYV and CABMV; Table S3. List of passion fruit plants evaluated in this study, symptoms observed, and virus identified; Table S4. List of green manure or spontaneous plants positive for CABYV and CABMV, and symptoms observed; Table S5. Pairwise nucleotide (nt) and amino acid (aa) sequence percentage identities generated with SDT for the complete genome and proteins P0 to P5 and P3a of CABYV-PF and other CABYV sequences from GenBank; Figure S1. RT-PCR and Southern blot hybridization detection of CABYV in passion fruit plants. RT-PCR positive control: Ctrl+ (RNA from pool PM2Ba). RT-PCR negative control: Ctrl- (H2O); Figure S2. CABMV detection by RT-PCR in all passion fruit plants (a), and in green manure/spontaneous plants CABYV-positives (b). RT-PCR positive control: Ctrl+ (RNA from pool PM2Ba). RT-PCR negative control: Ctrl- (H2O); Figure S3. CABYV detection in green manure/spontaneous plants from Lençóis by RT-PCR and Southern blot hybridization. RT-PCR positive control: Ctrl+ (RNA total from pool PM2Ba). RT-PCR negative control: Ctrl- (H2O); Figure S4. Recombination analysis among Brazilian CABYV isolates. (a) Schematic diagram showing putative recombinant fragments of Brazilian recombinant isolates from melon and CABYV-PF detected in RDP4. (b) Details of recombination events detected by RDR4 software in the genomes of CABYV-Brazilian melon recombinant isolates. Methods: R *=* RDP, G *=* GENECONV, B *=* Bootscan, M *=* MaxChi, C *=* Chimaera, S *=* SiScan, T *=* 3Seq.
