# Supplementary material for: Characterization of Cucurbit Aphid-Borne Yellows Virus (CABYV) from Passion Fruit in Brazil: Evidence of a Complex of Species within CABYV Isolates
Source: Viruses. 2023 Feb 1;15(2):410. doi: 10.3390/v15020410 (PMC9965994; doi:10.3390/v15020410)
Supplement: Supplementary file 1 [file viruses-15-00410-s001.zip › Vidal et al-Supplem.FigureS1-S4.pdf]

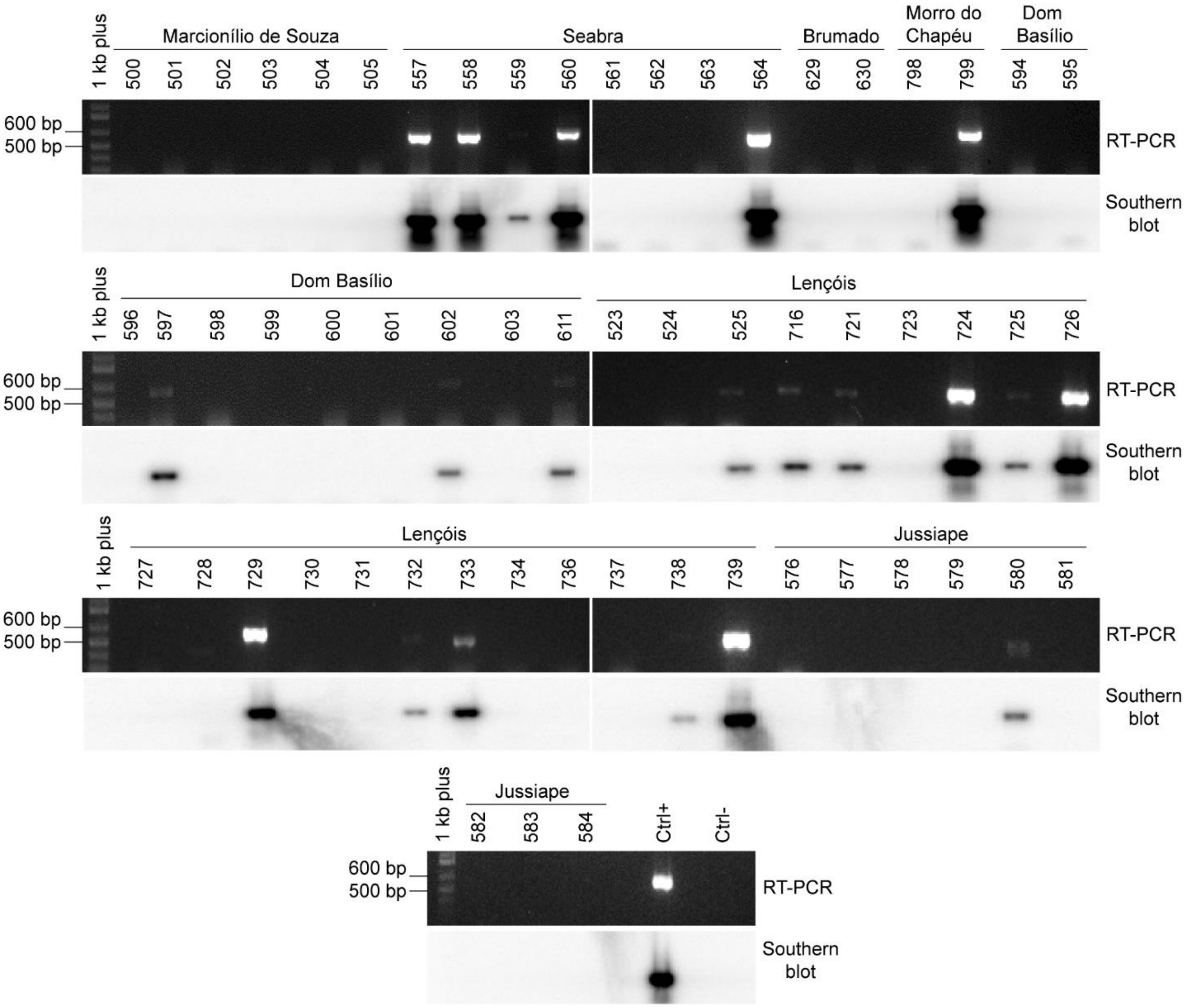

Figure S1. RT-PCR and Southern blot hybridization detection of CABYV in passion fruit plants. RT-PCR positive control: Ctrl+ (RNA from pool PM2Ba). RT-PCR negative control: Ctrl- (H_2O).

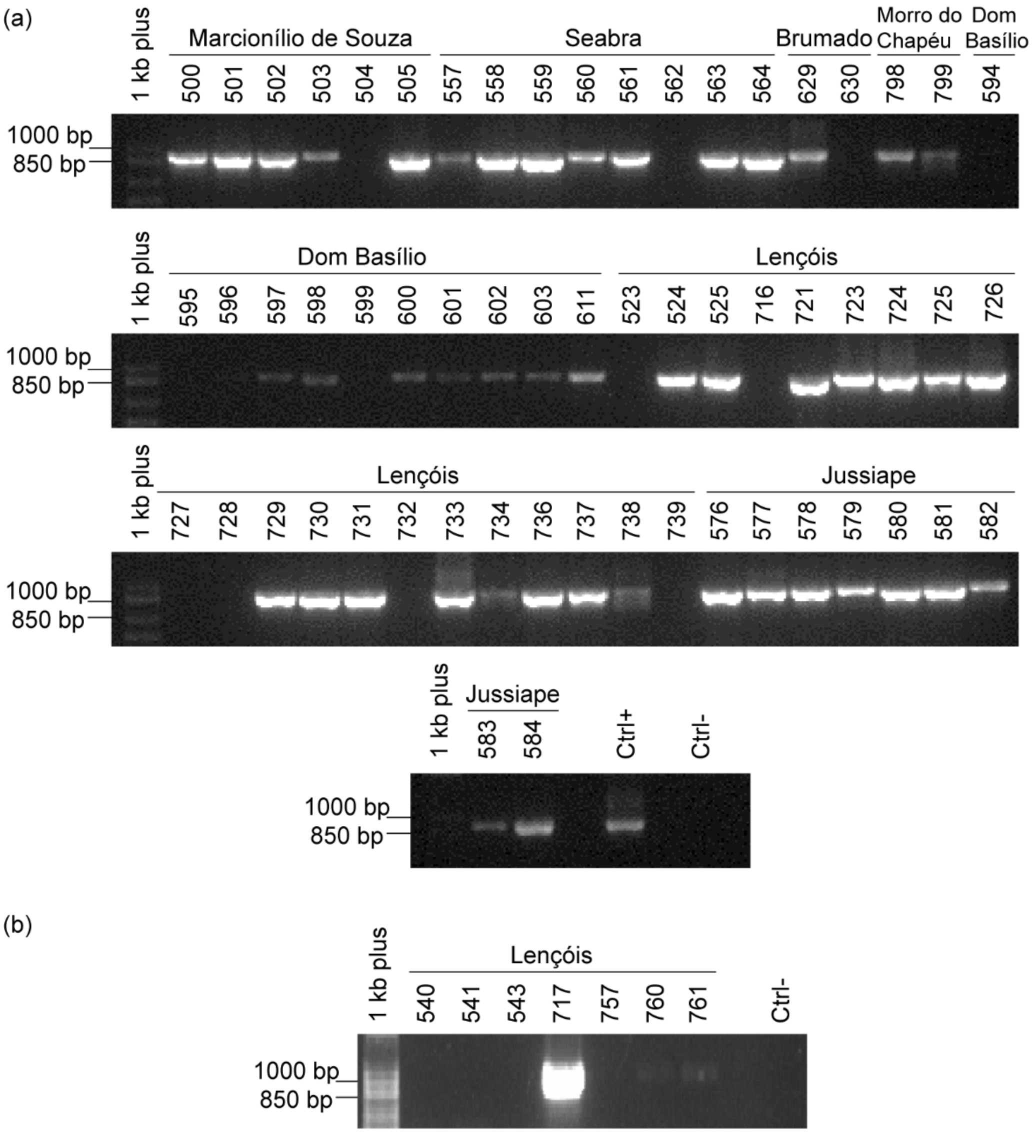

Figure S2. CABMV detection by RT-PCR in all passion fruit plants (a), and in green manure/spontaneous plants CABYV-positives (b). RT-PCR positive control: Ctrl+ (RNA from pool PM2Ba). RT-PCR negative control: Ctrl- (H_2O).

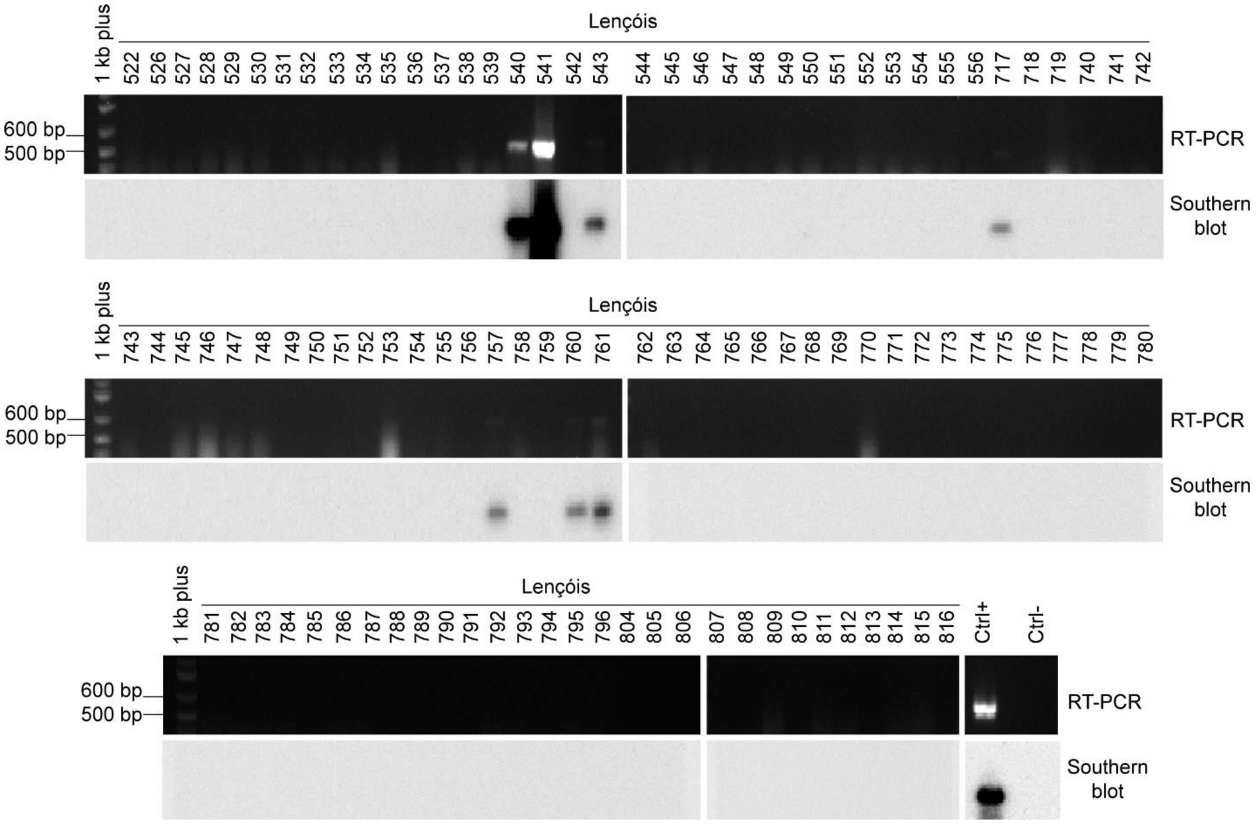

Figure S3. CABYV detection in green manure/spontaneous plants from Lençóis by RT-PCR and Southern blot hybridization. RT-PCR positive control: Ctrl+ (RNA total from pool PM2Ba). RT-PCR negative control: Ctrl- (H_2O).

(a)

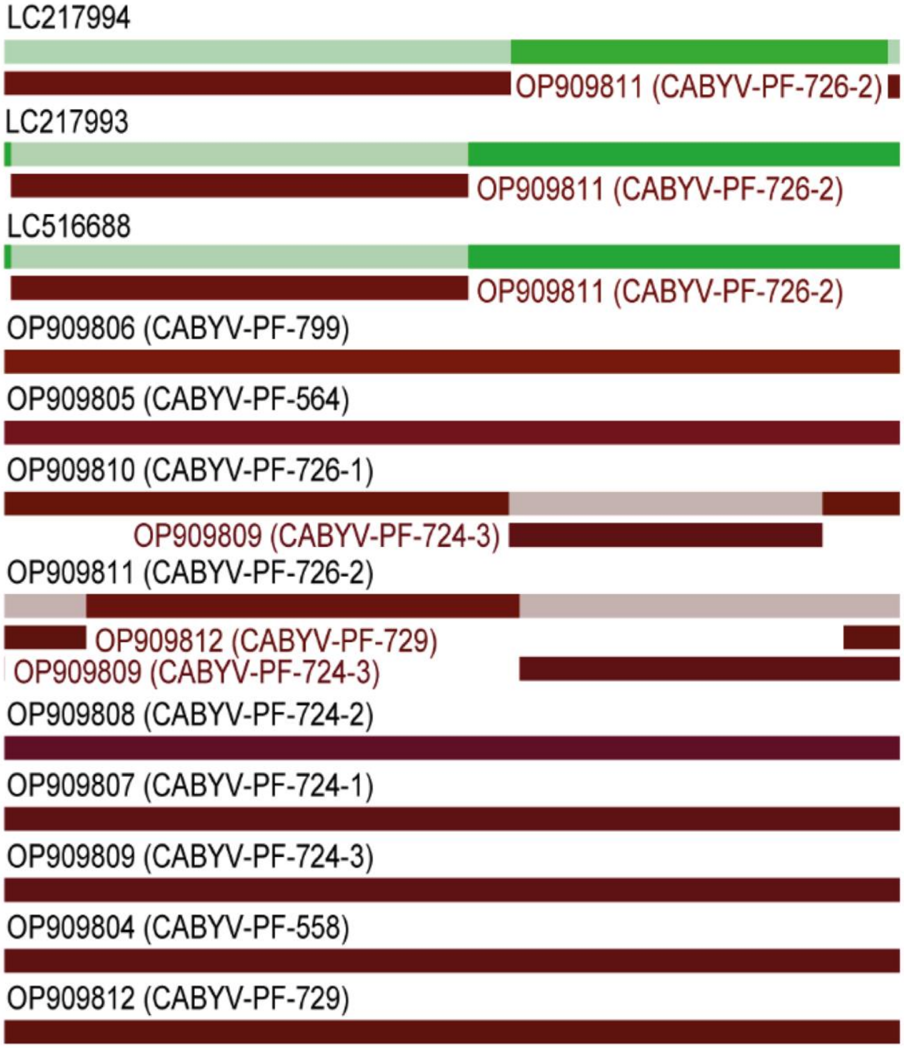

(b)

Sequence	Recombination breakpoint (nt)	Average p-Value (RDP)	Method of detection						
			R	G	B	M	C	S	T
LC217994	1-3347	1.12 X 10-73	+	+	+	+	+	+	+
	5673-5707	1.12 X 10-73	+	+	+	+	+	+	+
LC217993	50-3064	1.12 X 10-73	+	+	+	+	+	+	+
LC516688	40-3064	1.12 X 10-73	+	+	+	+	+	+	+

Figure S4. Recombination analysis among Brazilian CABYV isolates. (a) Schematic diagram showing putative recombinant fragments of Brazilian recombinant isolates from melon and CABYV-PF detected in RDP4. (b) Details of recombination events detected by RDR4 software in the genomes of CABYV-Brazilian melon recombinant isolates. Methods: R = RDP, G = GENECONV, B =Bootscan, M = MaxChi, C = Chimaera, S = SiScan, T = 3Seq.
