# Supplementary material for: Characterization of Cucurbit Aphid-Borne Yellows Virus (CABYV) from Passion Fruit in Brazil: Evidence of a Complex of Species within CABYV Isolates
Source: Viruses. 2023 Feb 1;15(2):410. doi: 10.3390/v15020410 (PMC9965994; doi:10.3390/v15020410)
Supplement: Supplementary file 1 [file viruses-15-00410-s001.zip › Vidal et al-Supplem.Table1-4.pdf]

Table S1. Primers used in this study.

Target	Primer name	Sequence 5′- 3′	Gene (bp)	Reference
CABYV	Modified-CE-9F	ATGAATACGGTCGCGGCTAGAAATC	CP/MP 600	This study
	CE-10R	CTATTTCGGGTTCTGGACCTGGC		[32]
CABMV	CABMVLNJP2492F	GGTTCGTGATGTTTTGGTGCC	HC-Pro/p3 ~900	[13]
	CABMVLNJP3373R	CAAAAAGCACGCACTCACAAATC		[13]
rbcL	SI_For	ATGTCACCACAAACAGAGACTAAAGC	rbcL ~550	[35]
	SI_Rev	GTAAAATCAAGTCCACCRCG		[35]
matK	KIM 3F	CGTACAGTACTTTTGTGTTTACGAG	matK ~850	[35]
	KIM 1R	ACCCAGTCCATCTGGAAATCTTGGTTC		[35]
5′Race GSP 1	AAP	GGCCACGCGTCGACTAGTACGGGIIGGGIIGGGIIG	5′ end ~700	[39]
	CABYVRACE581R	AAAGCGAGGCGAGTGTAAGC		This study
5′Race GSP 2	AUAP	GGCCACGCGTCGACTAGTAC	5′ end ~400	[39]
	CABYVRACE300R	GGCGGGCAGTCGTAAATCA		This study
3′Race GSP 1	M10	AAGCAGTGTTATCAACGCAGA	3′ end ~700	[39]
	CABYVRACE5063F	GCGTCTACAAGCTCTCGTCTC		This study
3′Race GSP 2	M10	AAGCAGTGTTATCAACGCAGA	3′ end ~350	[39]
	CABYVRACE5365F	CGCACAGTCAGAAAAACTCGC		This study
3′Race	M10PacIT50VN	AAGCAGTGTTATCAACGCAGATTAATTAAT50VN	cDNA	[39]
Amplicon1	CABYV5F	ACAAAAGATACGAGCGGGTGATG	~1100	This study
	CABYV1186R	TTGCTTAACACAGATGCATGAGTTT		This study
Amplicon2	CABYV377F	GATACAACGAAACCAGACAAACCTT	~3700	This study
	CE-10R	CTATTTCGGGTTCTGGACCTGGC		[32]
Amplicon3	Modified-CE-9F	ATGAATACGGTCGCGGCTAGAAATC	~2100	This study
	CABYV3R	ACACCGAAACGCCAGGGGG		This study

GSP: Gene-Specific Primer, AAP: Abridged Anchor Primer, AUAP: Abridged Universal.

Table S2. Number of passion fruit plants positive for CABYV and CABMV.

Sampling location	Nº of positive plants		
**Plant species**	**CABYV**	**CABMV**	**1 CABYV+CABMV**
Marcionílio de Souza			
Passiflora edulis	0/6 (0.0)2	5/6 (83.3%)	0/6 (0.0)
Seabra			
Passiflora edulis	5/8 (62.5%)	7/8 (87.5%)	5/8 (62.5%)
Brumado			
Passiflora edulis	0/2 (0.0)	1/2 (50%)	0/2 (0.0)
Morro do Chapéu			
Passiflora edulis	1/2 (50%)	2/2 (100%)	1/2 (50%)
Dom Basílio			
Passiflora edulis	3/11 (27.2%)	8/11 (72.7%)	3/11 (27.2%)
Lençóis			
Passiflora cincinnata	01/03	02/03	01/03
Passiflora alata	02/03	03/03	02/03
Passiflora edulis	8/15	10/15	05/15
Total	11/21 (52.3%)	15/21 (71.4%)	8/21 (38%)
Jussiape			
Passiflora edulis	1/9 (11.1%)	9/9 (100%)	1/9 (11.1%)

[1] Co -infection.

[2] Percentage of infected plants is in parenthesis

Table S3. List of passion fruit plants evaluated in this study, symptoms observed, and virus identified.

City	Code	Species	Symptoms	Virus
Marcionílio de Souza	500	*Passiflora edulis*	CR, B, M	CABMV
	501	*Passiflora edulis*	M	CABMV
	502	*Passiflora edulis*	M	CABMV
	503	*Passiflora edulis*	M	CABMV
	504	*Passiflora edulis*	M, YS, B, CR, LD	Not detected
	505	*Passiflora edulis*	M, YS, LD	CABMV
Seabra	557	*Passiflora edulis*	M, C, LD	CABYV, CABMV
	558	*Passiflora edulis*	M, C, LD	CABYV, CABMV
	559	*Passiflora edulis*	M, C, LD	CABYV, CABMV
	560	*Passiflora edulis*	M, C, LD	CABYV, CABMV
	561	*Passiflora edulis*	M, C, LD	CABMV
	562	*Passiflora edulis*	M, C, LD	Not detected
	563	*Passiflora edulis*	M, C, LD	CABMV
	564	*Passiflora edulis*	M, C, LD	CABYV, CABMV
Brumado	629	*Passiflora edulis*	M, B, LD	CABMV
	630	*Passiflora edulis*	LD	Not detected
Morro do Chapéu	798	*Passiflora edulis*	M, B	CABMV
	799	*Passiflora edulis*	M	CABYV, CABMV
Dom Basílio	594	*Passiflora edulis*	Y, LD	Not detected
	595	*Passiflora edulis*	M	Not detected
	596	*Passiflora edulis*	M, VB	CABMV
	597	*Passiflora edulis*	M	CABYV, CABMV
	598	*Passiflora edulis*	M	CABMV
	599	*Passiflora edulis*	IS	Not detected
	600	*Passiflora edulis*	LD, IS	CABMV
	601	*Passiflora edulis*	LD, M, Y	CABMV
	602	*Passiflora edulis*	IS, M	CABYV, CABMV
	603	*Passiflora edulis*	YD, LD	CABMV
	611	*Passiflora edulis*	Y, LD, M	CABYV, CABMV
Lençóis	523	*Passiflora cincinnata*	M, LD	Not detected
	524	*Passiflora cincinnata*	M	CABMV
	525	*Passiflora cincinnata*	M	CABYV, CABMV
	716	*Passiflora edulis*	VB	CABYV
	721	*Passiflora edulis*	GS, M, B	CABYV, CABMV
	723	*Passiflora edulis*	B, LD, M	CABMV
	724	*Passiflora edulis*	B, LD, M	CABYV, CABMV

	725	*Passiflora edulis*	B, LD, M	CABYV, CABMV
	726	*Passiflora edulis*	B, LD, M	CABYV, CABMV
	727	*Passiflora edulis*	M, VB	Not detected
	728	*Passiflora edulis*	B, LD, M	Not detected
	729	*Passiflora edulis*	B, LD, M	CABYV, CABMV
	730	*Passiflora edulis*	B, LD, M	CABMV
	731	*Passiflora alata*	B, M	CABMV
	732	*Passiflora edulis*	B, LD, M	CABYV
	733	*Passiflora alata*	B, LD, M	CABYV, CABMV
	734	*Passiflora edulis*	B, LP	CABMV
	736	*Passiflora edulis*	B, LD	CABMV
	737	*Passiflora edulis*	M, B, YS	CABMV
	738	*Passiflora alata*	M, B, VB	CABYV, CABMV
	739	*Passiflora edulis*	B, LD, M	CABYV
Jussiape	576	*Passiflora edulis*	VB, B, Y	CABMV
	577	*Passiflora edulis*	B, LD, M, YS	CABMV
	578	*Passiflora edulis*	B, LD, FD	CABMV
	579	*Passiflora edulis*	B, LD, VW	CABMV
	580	*Passiflora edulis*	M, VW	CABYV, CABMV
	581	*Passiflora edulis*	M, LD, Y	CABMV
	582	*Passiflora edulis*	M, LD, Y	CABMV
	583	*Passiflora edulis*	B, LD, M	CABMV
	584	*Passiflora edulis*	M, N	CABMV

Abbreviation of symptoms: Crinkled (CR), mosaic (M), leaf deformation (LD), fruit deformation (FD), Blistering (B), yellow spot (YS), chlorosis (C), yellowing (Y), insect damage (ID), vein banding (VB), green spot (GS), vein whitening (VW), leaf purplish (LP).

Table S4. List of green manure or spontaneous plants positive for CABYV and CABMV, and symptoms observed.

Code	Family/ Genus	Species	Symptoms	Virus
540	Fabaceae/ *Macroptilium*	*Macroptilium* spp.	M	CABYV
541	Cucurbitaceae/ *Cucumis*	*Cucumis* spp.	Y	CABYV
543	Fabaceae/ *Macroptilium*	*Macroptilium* spp.	VB	CABYV
717	Fabaceae/ *Macroptilium*	*Macroptilium* spp.	UR	CABYV, CABMV
757	Fabaceae/ *Stylosanthes*	*Stylosanthes* spp.	YS	CABYV
760	Malvaceae/ *Sida*	*Sida* spp.	Y	CABYV, CABMV
761	Bignoniaceae/ *Bignonia*	*Bignonia* spp.	AS	CABYV, CABMV

Symptoms: mosaic (M), yellow spot (YS), chlorosis (C), yellowing (Y), vein banding (VB), asymptomatic (AS), and unregistered (UR).
